# Predicting Acute Pancreatitis Severity: Comparison of Prognostic Scores

**DOI:** 10.4021/gr364w

**Published:** 2011-09-20

**Authors:** Marco Simoes, Patricia Alves, Helder Esperto, Catarina Canha, Elisa Meira, Erica Ferreira, Manuel Gomes, Isabel Fonseca, Benilde Barbosa, Jose Nascimento Costa

**Affiliations:** a Internal Medicine Department, Hospitais da Universidade de Coimbra, Portugal

**Keywords:** Acute Pancreatitis, Prognostic Scores, APACHE II, Ranson, Glasgow, Balthazar, C-reactive Protein

## Abstract

**Background:**

Acute pancreatitis has a broad clinical spectrum, from mild illness to multiple organ failure and death. Prognostic scores have been developed or adapted to predict disease severity. This study aimed to compare the prognostic scores according to sensitivity and specificity, receiver operating characteristic curves and area under the curve. Statistical correlation with disease severity, length of hospital stay, mortality and complication rates.

**Methods:**

Retrospective analysis of the clinical data of patients admitted to an Internal Medicine ward with the diagnosis of acute pancreatitis over a ten year period. Evaluation of prognostic scores: Ranson, Glasgow-Imrie, Balthazar, APACHE II (admission and at 48 hours) and C-reactive protein (48 hours), was carried out as well as statistical analysis using Microsoft Excel 2007® and SPSS 16®. The confidence interval used was 95%.

**Results:**

Data from 193 clinical files was collected. However, 67 were excluded due to lack of information. According to the Atlanta criteria, 90 cases were deemed as mild and 36 severe. The mortality rate was 6% and the local complication rate was 9.3%. Ranson, Glasgow and APACHE II scores had significant correlation with mortality. Apart from C-reactive protein levels at 48 hours, all scores had significant correlation with disease severity. The scores with best area under the curve correlation were APACHE II (48 hours): 0.892, Ranson: 0.879, and APACHE II (admission): 0.861.

**Conclusions:**

The most accurate prognostic scores in this study were APACHE II (48 hours) and Ranson. APACHE II at admission was a good indicator, impaired only by high false positive ratio.

## Introduction

Acute pancreatitis (AP) diagnostic criteria and outcome prediction were the subject of discussions over the years, but the Atlanta Symposium in 1992 set the standards relating diagnostic criteria and disease severity [1]. Predicting severity is an essential step while evaluating a patient with AP as it allows physicians to stratify disease severity and management strategies [2, 3]. Several prognostic scoring systems based of clinical, laboratorial and radiologic evaluations have been created or adapted to predict outcome, some based on local complications and other reflecting systemic manifestations of AP. Ranson’s score [4] is possibly the most used scoring system created specifically for AP. The Acute Physiology And Chronic Health Evaluation II (APACHE II) [5] scoring system was created to evaluate any severe acute illness and has successfully been used to predict AP severity. Unspecific biomarkers, such as C-reactive protein (CRP) have also been studied as outcome predictors, but it has only been useful for predicting complications, namely necrotizing AP [2, 6].

This study aimed to compare specific, unspecific and morphological based prognostic scoring systems regarding disease severity, according to sensitivity and specificity, receiver operating characteristic curve and area under the curve. Correlation with length of hospital stay, mortality and complication rates was determined in order to evaluate which scores were better predictors of these outcomes.

## Methods

The authors carried out a retrospective analysis of the clinical data of patients admitted with AP to an Internal Medicine ward over a period of ten years. Demographic features, analytical and radiological findings, disease severity as well as final outcome were evaluated. Between 1997 and 2006, 193 patients with AP were admitted to our ward, but 67 were excluded due to insufficient information on clinical files. Ranson, Glasgow and APACHE II’s (at admission and at 48 hours) prognostic scores were calculated in all patients. CRP at 48 hours was evaluated in all patients. Balthazar’s scores were calculated only when a CT scan was performed (48 patients). Statistical correlation between these scoring systems and disease severity as defined by the Atlanta criteria [1], mortality and complication rates, as well as length of hospital stay was carried out with confidence interval of 95%.

### Definitions

The diagnostic criteria for AP were those defined by the 2006 AP Guidelines [2], as the presence of at least two of the following features: 1) characteristic abdominal pain; 2) elevation over 3 times the upper normal limit of serum amylase/lipase; 3) characteristic features on computer tomography (CT) scan. Over the last twenty years much has been learned about this condition and therapeutic strategies and imaging techniques have improved. In many studies the criteria adopted vary, occasionally reflecting local experience or national guidelines [7]. In this study severe AP was diagnosed according strictly to Atlanta criteria [1] (Table 1). Local complications were determined by CT scan, which was performed only when clinical course was unfavorable, when morphologic changes were detected on transabdominal ultrasonography or based on clinical suspicion.

**Table 1:** Severe Acute Pancreatitis Criteria

Diagnose of severe AP	Criteria
Early Prognostic Scores	Ranson ≥ 3
	APACHE II ≥ 8
Organ Failure	Systolic pressure < 90 mmHg
	PaO_2_ ≤ 60 mmHg
	Creatinine > 2.0 mg/L after rehydratation
	Gastrointestinal bleeding > 500 cc/24 h
Local Complications (on CT scan)	Necrosis
	Abcess
	Pseudocyst

### Ranson

Originally Ranson criteria were created for alcohol-induced AP [4] and were revised in 1979 for gallstone-induced AP [8]. Original Ranson score was used by default as alcohol-induced AP was the most prevalent etiology. When gallstones were found, revised Ranson score was used. The cutoff value accepted in the literature is 3 [2, 6, 9-12].

### Glasgow-Imrie

Also known as Glasgow score, it includes eight laboratory criteria and age. Like Ranson, this scoring system can only be calculated at 48 hours. The cutoff value used is also 3 [6, 11, 13].

### Balthazar

In 2002 Balthazar created a severity stratification method based on necrosis extent and pancreatic morphologic changes [14]. This scoring system has a maximum of ten points, and patients with scores higher than 6 have a higher rate of complications and death [6, 11, 15, 16].

## APACHE II

The Acute Physiology and Chronic Health Evaluation II scoring system was created in 1985 to evaluate any severe acute disease in an Intensive Care Unit setting [2, 5, 6, 11, 12]. Although it was not specifically created to evaluate AP severity, it has been successfully used to predict AP outcome. In this context, APACHE II was included by the Atlanta Symposium, with a cutoff value of 8 [1].

### C-reactive protein

CRP is an acute phase reactant synthesized by the liver, with peak serum values occurring within the first 72 hours after symptoms onset [6]. Severity stratification by CRP has been used due to its availability and cost. The late serum peak impairs its utility as a biomarker on admission. Nevertheless, CRP serum level over 15 mg/dL at 48 hours is a good indicator of necrotizing pancreatitis [2, 6, 15, 17, 18].

### Statistics

Results are expressed as mean ± standard deviation. Statistical analysis includes Student t-test, Fisher exact test, Pearson’s chi-square test, McNemars test, Mann-Whitney test and Odds ratio. P values less than 0.05 were considered statistically significant. Receiver Operating Characteristic (ROC) curves and Area Under the Curve (AUC) analysis were used to compare prognostic scoring systems. Data was gathered using Microsoft Excel 2007® and analyzed by SPSS 16.0®.

## Results

### Clinical data

**Figure 1:**
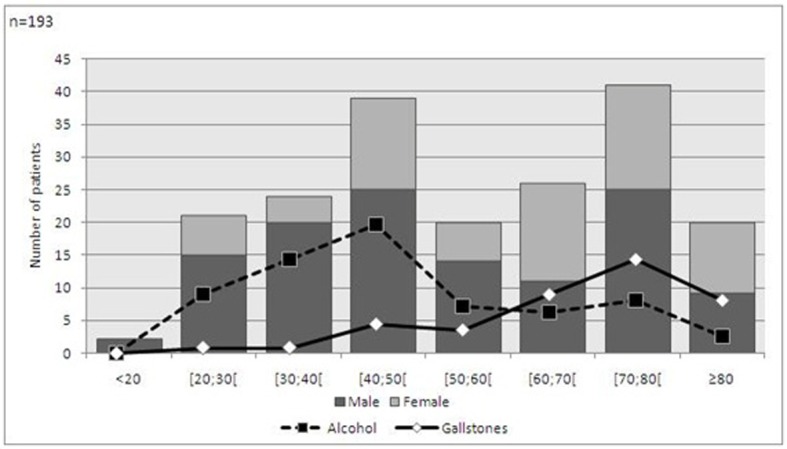
Demographic features and etiology of acute pancreatitis.

Clinical data was collected in 193 cases. In our series there was clear male prevalence, with a 1.7:1 ratio. The mean age was of 52.42 years (± 19.62) in males and 60.31 (± 19.60) in females. Based on age, there were two peak incidences: between 40 and 50 years and between 70 and 80 years. The most common etiology was alcohol consumption (39.3%), followed by gallstones (24.1%). In 31.9% no identifiable cause was found, but in some cases microlithiasis was suspected. Relating age class, gender and etiology, we could find two patient profiles: the middle-aged male patient with a history of alcohol consumption and the elderly woman with gallstones (Fig. 1).

CT scan was carried out in 48 patients, half had pancreatic morphological changes and 17% also had necrosis. Local complications were found in 18 patients, the most common being pancreatic pseudocyst (56.5%) and aseptic necrosis (30.4%). In our series the mortality rate was 5.7% (11 patients), similar to what is reported by most studies [2]. The mean length of hospital stay was of 10.78 days (± 7.93).

Due to unavailable data, disease severity was determined retrospectively in only 126 patients, 29% of which had severe AP. Patients with severe AP were notably older, 69.89 years (± 17.43) than those with mild disease, 52.06 years (± 19.73). All deaths occurred in patients with severe AP.

### Prognostic scores

#### Ranson

**Figure 2:**
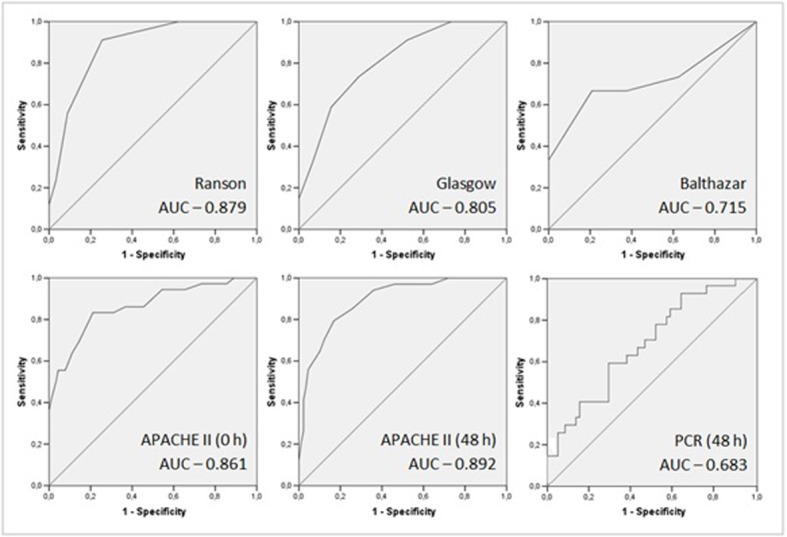
ROC curves and AUC of acute pancreatitis prognostic scores.

The authors found a sensitivity of 91.2% and specificity of 74.4% related to degree of severity, achieving a good discriminatory ability with AUC of 0.879 (0.818-0.940) (Fig. 2). The high negative predictive value (NPV), 95.7%, allows this score to exclude severe AP outcome (Table 2).There was significant correlation between disease severity and Ranson score 3 or above, with odds ratio of 30.131 (8.401-107.857, P < 0.001).

**Table 2:** Value of Prognostic Scores in the Prediction of Severe Acute Pancreatitis

	Sensitivity (%)	Specificity (%)	PPV (%)	NPV (%)	AUC
Ranson	91.2	74.4	57.4	95.7	0.879 (0.818-0.940)
Glasgow	73.5	71.1	49.0	87.7	0.805 (0.724-0.886)
Balthazar	26.7	100.0	100.0	68.6	0.715 (0.528-0.902)
APACHE II (0 h)	83.3	68.9	51.7	91.2	0.861 (0.785-0.938)
APACHE II (48 h)	79.4	83.1	64.3	91.4	0.892 (0.832-0.953)
CRP (48 h)	44.4	70.7	41.4	73.2	0.683 (0.564-0.802)

PPV: Positive predictive value; NPV: negative predictive value; AUC: Area under the curve.

Regarding mortality, as there were no deaths in the group with Ranson score less than 3, odds ratio was impossible to determine. However, by Fisher’s exact test there was significant evidence that there was increased mortality in the group with Ranson score equal or above 3 (P = 0.001). There was no statistical difference regarding complication rates between the groups (P = 0.171). Concerning length of hospital stay, there was significant difference between the medians by the Mann-Whitney test (P = 0.016) (Table 3).

**Table 3:** Correlation of Prognostic Scores With Study Endpoints

	Severity	Mortality	Complications	Length of Stay
Ranson	30.101 (8.401-107.857)^a^ P < 0.001^b^	*** P = 0.001^c^	2.045 (0.723-5.786)^a^ P = 0.171^b^	8 versus 12 days P = 0.016^d^
Glasgow	6.838 (2.814-16.615)^a^ P < 0.001^b^	*** P = 0.001^c^	1.323 (0.473-3.698)^a^ P = 0.593^b^	8 versus 12 days P = 0.003^d^
Balthazar	*** P = 0.017^c^	14.333 (0.707-290.431)^a^ P = 0.161^c^	*** P = 0.012^c^	17 versus 37 days P < 0.001^d^
APACHE II (0 h)	11.071 (4.140-29.605)^a^ P < 0.001^b^	*** P < 0.001^c^	1.049 (0.377-2.921)^a^ P = 0.927^b^	8 versus 11 days P = 0.029^d^
APACHE II (48 h)	19.029 (7.003-51.701)^a^ P < 0.001^b^	*** P < 0.001^c^	1.882 (0.668-5.304)^a^ P = 0.227^b^	9 versus 13 days P = 0.010^d^
CRP (48 h)	1.929 (0.749-4.972)^a^ P = 0.171^b^	2.375 (0.559-10.086)^a^ P = 0.252^c^	6.600 (2.090-20.843)^a^ P = 0.001^c^	7 versus 13,5 days P < 0.001^d^

*** Odds ratio not calculated; a: Odds Ratio; b: Chi-square test; c: Fisher exact test; d: Mann-Whitney test.

#### Glasgow-Imrie

In this case series this score was slightly inferior to Ranson, as sensitivity was 74.5% and specificity 71.1% (Table 2). This score also had good discriminatory ability, as the AUC was 0.805 (0.724-0.886) (Fig. 2).

Significant correlation with disease severity was found, with odds ratio of 6.838 (2.814-16.615, P < 0.001). As in the Ranson score analysis, as there were no deaths in one of the groups, odds ratio was impossible to determine. There were differences between groups by Fisher exact test, with increased mortality in the group with Glasgow score equal or above 3 (P = 0.001). There was no statistical difference between groups regarding complication rates (P = 0.593). The median length of stay was significantly superior in those patients with at least 3 Glasgow criteria (P = 0.003) (Table 3).

#### Balthazar

CT scan was performed only in 48 patients, based on clinical evaluation and suspicion of complications. Therefore our results may be biased by pretest probability and small sample size (only 4 patients in the group with higher score). Using 6 as cutoff value, the specificity was 100% but with very low sensitivity, namely 26.7% (Table 2). With AUC of 0.715 (0.528-0.902), discrimination ability was only passable (Fig. 2).

As there were no severe cases of AP in the group with lower Balthazar score, odds ratio was not determined. However, Fisher’s exact test showed that the group with the higher Balthazar score included more severe cases (P = 0.017). Concerning mortality, there was no significant difference between groups (P = 0.161), but odds ratio lacks precision due to sample size. There was significant correlation with complication rate (Fisher exact test, P = 0.012), but odds ratio was not determinable. There was significant difference between length of stay medians as calculated by the Mann-Whitney test (P < 0.001) (Table 3).

#### APACHE II

Although not created specifically for risk stratification in AP, the APACHE II score at admission and at 48 hours had a good discriminatory ability. Using 8 as cutoff value, the AUC at admission was 0.861 (0.785-0.938), increasing to 0.892 (0.832-0.953) at 48 hours (Fig. 2). PPV, NPV and specificity also improved over time. Sensitivity decreased slightly at 48 hours (83.3% to 79.4%) (Table 2).

There was significant correlation with disease severity. At admission the odds ratio was 11.071 (4.140-29.605, P < 0.001) and at 48 hours was 19.029 (7.003-51.701, P < 0.001). Regarding mortality, as there were no deaths in patients with APACHE II lower than 8, the odds ratio was not possible to determine. However, Fisher exact test showed significant correlation in both evaluations (P < 0.001). No significant association between APACHE II score and complication rate was found (P = 0.927; P = 0.227). Length of hospital stay correlated with APACHE II score, at admission (P = 0.029) and at 48 hours (P = 0.003) (Table 3).

#### C-reactive protein

In our study CRP value was not a good marker for disease severity (P = 0.171) or mortality (P = 0.252). However, with the exception of Balthazar score, this was the only score capable of predicting the occurrence of local complications, with odds ratio of 6.600 (2.090-20.843, P = 0.001). Hospital length of stay was also associated with higher CRP values at 48 hours (P < 0.001) (Table 2).

Regarding the prediction of severity, CRP had a low sensitivity (44.4%) and specificity (70.7%) (Table 3) and its discriminatory ability was poor, with AUC: 0.683 (0.564-0.802) (Fig. 2).

## Discussion

Prognostic scores were created or adapted in AP to predict disease severity. In this context, APACHE II and Ranson scores were the most accurate among those evaluated. This finding was concordant with several previous studies [2, 6, 18].

APACHE II (48 hours) had the most powerful specificity and sensitivity, but Ranson had the best negative predictive value. The main drawback of Ranson score is the time interval required for its calculation. APACHE II score at admission were slightly less reliable, but proved to be a useful screening score, with very good negative predictive values. The NPV and PPV values obtained are similar to those described by Chatzicostas et al [19], but in the present study no statistical difference was found between Ranson and APACHE II AUC’s values. Different APACHE II cutoff values were used in our study as compared to Chatzicostas’s, thus explaining this disparity. The cutoff value used for APACHE II score was based on American College of Gastroenterology guidelines [2] and the Atlanta Symposium [1]. Naturally, when APACHE II score was repeated in subsequent days, specificity and positive predictive values improved. Therefore, serial evaluation of APACHE II’s score may prove to be valuable regarding disease severity and clinical outcome, with direct consequences on the level of monitoring and management of patients with AP. The Glasgow-Imrie score was inferior to both Ranson and APACHE II, therefore its usefulness is questionable.

The results obtained for Balthazar score was as expected according to evidence described in literature [2, 14]. However, in our analysis Balthazar score results may have been biased by several factors: small sample size, small number of subjects in one of the subgroups and the pretest probability, as CT scan was only performed in selected patients based on clinical evolution. Consequently, the authors cannot present valid conclusions regarding this prognostic score. In order to obtain reliable data, a prospective study should be done, with CT scan performed in all patients, regardless of clinical evaluation and prognostic scores. Gürleyik et al [15], presented a small prospective study with these characteristics and concluded that Balthazar has a better accuracy than APACHE II scores. Very high accuracy in disease severity prediction was shown by Balthazar when this scoring system was presented [14].

Other imaging techniques, as magnetic resonance imaging (MRI) and endoscopic ultrasound (EUS), have improved significantly over the past years and have become increasingly available. Several studies showed potential for the usage of these techniques on the evaluation of AP. CT scan usage is impaired by the usage of radiation and the risk of contrast induced nephrotoxicity. MRI does not have these problems and has proved to be superior in evaluation of mild AP, peripancreatic fat infiltration and pancreatic and biliary ducts assessment. There is significant concordance between MRI and CT scan, as well as with clinical course. However, no correlation was found with APACHE II, therefore MRI has limited role in determining systemic complications [20]. Several MRI protocols can be used in order to increase diagnostic accuracy [21]. Transabdominal ultrasonography is not accurate in many cases due to overlying gas and retroperitoneal location of the pancreas. EUS has better resolution and can detect the presence of microlithiasis, occult pancreatic neoplasms and pancreas divisum, which can cause AP [22, 23]. The presence of peripancreatic edema in EUS is associated with disease severity [23], but more studies are needed to validate the role of EUS in the staging and prognosis of AP. In this study identifiable cause was found in almost one third of the patients, therefore EUS could have been useful.

CRP value at 48 hours was not a useful prognostic score for disease severity or mortality. However, in our sample it was the only score that predicted the occurrence of local complications with statistical significance (Balthazar score results were disregarded due to eventual bias). These findings were expected, as similar results had been previously described by Rau [3].

These stratification scores are adjunctive to each other. The authors suggest that the evaluation strategy of patients with AP should include APACHE II’s at admission, followed by daily reassessments. CRP must be determined at 48 hours as it can predict the occurrence of local complications. Based on APACHE II’s scoring system and CRP value, contrast-enhanced CT scan should be performed in selected patients, 48-72 hours post admission. EUS and MRI can also be used, when there is suspicion of microlithiasis or local complications. However, as their role is not yet perfectly defined, we cannot advise the routine usage of these techniques. Perhaps a revision of the Atlanta criteria is needed in order to reflect the evolution of knowledge and imaging techniques, as well as other prognostic scores that have been created.
